# Associations between cumulative exposure to potentially traumatic events and self-reported oral health in the Tromsø Study: Tromsø7

**DOI:** 10.1186/s12903-025-06005-2

**Published:** 2025-06-02

**Authors:** Hege Nermo, Jeppe Bjørnskov Goll, Simen Isaksen, Sigurd Eggen Trondsen, Kamilla Rognmo, Jens C. Thimm, Catharina Elisabeth Arfwedson Wang, Tiril Willumsen, Jan‑Are K. Johnsen

**Affiliations:** 1 The Public Dental Health Competence Center of Northern Norway, Tromsø, Norway; 2 Department of Clinical Dentistry, Faculty of Health Sciences, UiT The Arctic University of Norway, Tromsø, Norway; 3 Nordland Hospital Trust, Bodø, Norway; 4 Helgeland Hospital Trust, Mo i Rana, Norway; 5 Department of Psychology, UiT The Arctic University of Norway, Tromsø, Norway; 6 Department of Pediatric Dentistry and Behavioural Science, University of Oslo, Oslo, Norway

**Keywords:** Oral health, Dental health, Potentially traumatic events, Public health, Dental anxiety, Trauma-informed care

## Abstract

**Background:**

Potentially Traumatic Events (PTEs), such as accidents, childhood neglect or abuse, can affect mental and physical health. The study investigated the association between PTEs and self-reported oral health (SROH), focusing on cumulative exposure to multiple events, the types of events, and the timing of exposure.

**Methods:**

Data were collected from the seventh survey of the Tromsø Study, which invited all residents over 40 in Tromsø, Norway. A total of 21,069 participants took part, comprising 47,5% male and 52,5% female respondents, who completed assessments of PTEs and SROH. Chi-square (Χ²) tests evaluated unadjusted associations, and a series of logistic regression models were employed to investigate the association of PTEs with poor SROH, controlling for sociodemographic variables, emotional distress, and oral health-related behaviours.

**Findings:**

The likelihood of reporting poor SROH was higher among those who experienced more PTEs, and this relationship persisted after adjusting for sex, age, socioeconomic status, oral health-related behaviour, dental anxiety, emotional distress and adverse dental events. Subsequent analyses differentiated the associations by type and timing (before and after age 18) of events. Interpersonal events involve direct interactions with others (e.g., abuse, bullying), while impersonal events refer to broader circumstances (e.g., accidents, natural disasters). Adverse dental events, classified as impersonal events, demonstrated the strongest association with poor SROH. The associations between interpersonal events and poor SROH varied more depending on covariates than impersonal events. The association strengthened when adjusting for sociodemographic factors but weakened when accounting for oral health behaviours, dental anxiety, and emotional distress. Notably, impersonal events occurring before age 18 were consistently associated with poor SROH across all models.

**Conclusions:**

Experiencing multiple PTEs throughout life is associated with poor SROH. Among the various PTEs, adverse dental events showed the strongest association with poor SROH, emphasising the importance of addressing dental care’s emotional and psychological aspects, particularly in paediatric settings, to support long-term oral health outcomes.

**Clinical trial number:**

Not applicable.

## Introduction

### Background and rationale for the study

Oral diseases remain a global health challenge, mirroring other non-communicable diseases in their multifactorial nature [1]. Oral health is shaped by an interplay of biological, psychological, and social determinants [2]. These complex factors contribute to the unequal distribution of oral disease, with the burden disproportionately affecting underserved and marginalised groups, such as disadvantaged socioeconomic populations [3, 4], institutionalised older individuals [5], and individuals living with severe mental illnesses [6].

Potentially Traumatic Events (PTEs) are common across populations [7] and have been linked to a wide range of physical and mental health problems [8, 9]. However, as shown in global epidemiological studies, exposure to PTEs varies by sociodemographic factors and prior traumatic experiences [7], meaning that their accumulation is not random. For example, individuals facing economic hardship may be at greater risk of experiencing violence or neglect, further compounding disparities in health. The impact of PTEs can vary based on the type of event [10], the accumulation of multiple events [11], and the age at which exposure occurred [12]. Given these variations, it is important to consider the cumulative effects, the nature of the events, and the timing of exposure when examining their potential impact on health.

In the context of oral health, PTEs are particularly relevant due to their association with dental anxiety. Painful or negative dental experiences have long been recognised as an important part of the aetiology of dental anxiety [13], frequently leading to avoidance of care [14]. This avoidance can exacerbate oral diseases, increasing the need for more complex and potentially distressing treatments, thus reinforcing dental anxiety in a self-perpetuating cycle [15]. Additionally, PTEs outside the dental setting have been associated with dental anxiety in general populations [16, 17]. Among survivors of child sexual abuse, dental anxiety appears to be triggered by sensory stimuli reminiscent of past trauma, suggesting it is trauma-driven [18]. Research also connects adverse childhood experiences to altered pain perception, with trauma-exposed children having lower pain thresholds, greater pain sensitivity, and lower pain tolerance in adulthood [19, 20, 21]. These factors make individuals with a history of trauma more susceptible to pain and anxiety during dental procedures and more prone to developing dental anxiety.

PTEs, especially during childhood, can have long-lasting effects on oral health [22, 23]. However, the association of cumulative PTEs experienced over a lifetime, as well as the type and timing of these events, with oral health—particularly when considering emotional distress or dental anxiety—remains underexplored.

**Fig. 1:**
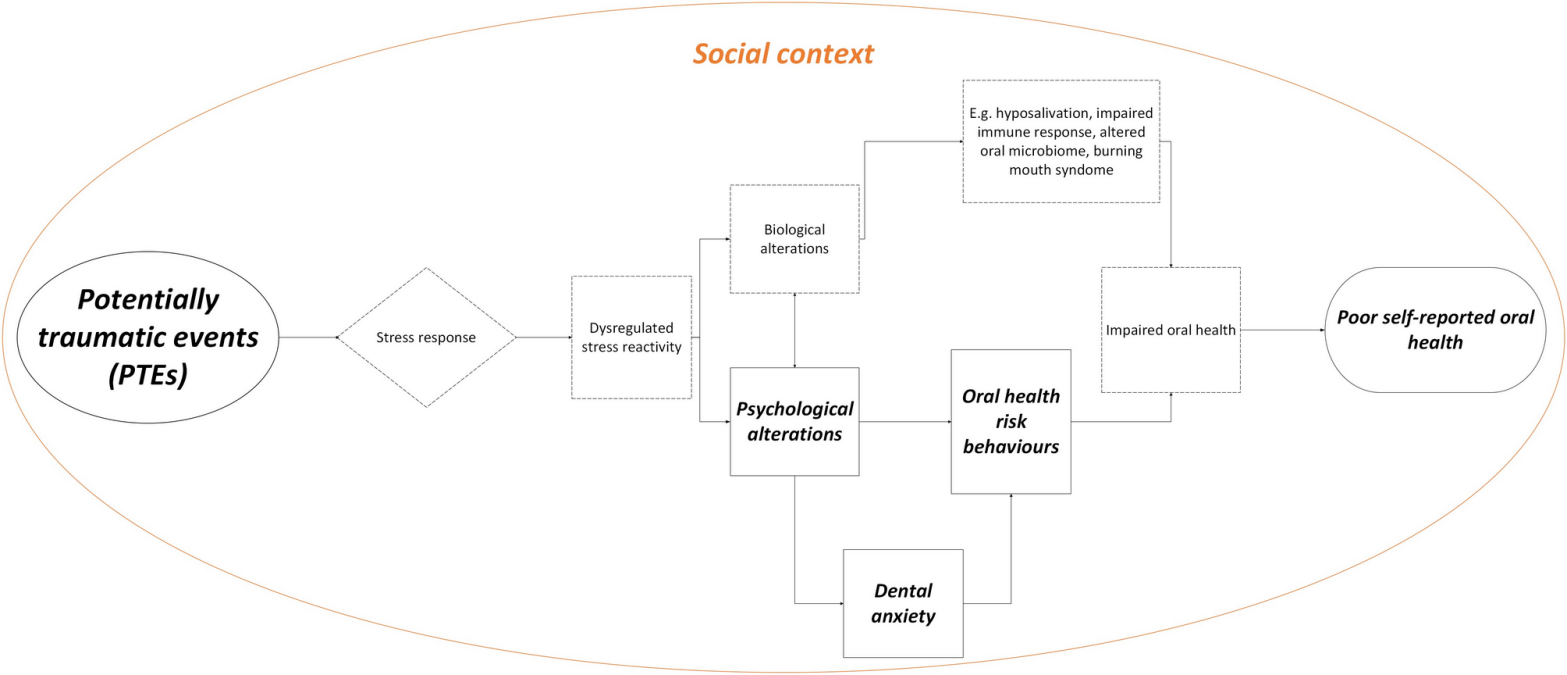
Conceptual Model Linking Potentially Traumatic Events to Poor SROH. This simplified conceptual model illustrates potential pathways linking PTEs to poor SROH. It is inspired by Cohen’s heuristic stage model of stress and disease [24], Schnurr’s model that relates traumatic exposure to physical health outcomes [25], and the biopsychosocial model of health and illness [26]. The purpose of the figure is to visualise how PTEs may contribute to poor oral health through dysregulated stress reactivity and subsequent psychological, biological, and behavioural alterations. For simplicity, the process is illustrated as one-way with various stages, although social context (including personal and cultural factors) forms the backdrop for these pathways. While the model highlights dysregulated stress reactivity as a key mechanism, it does not imply that it is the sole pathway to poor oral health, as other biological, social and behavioural determinants may also contribute to these outcomes. The pathways depicted here are not exhaustive, and bidirectional effects and feedback loops are not represented

To illustrate the theoretical framework underlying the potential associations between PTEs and oral health, one might look to established models from stress and disease research [24], trauma and physical health [25], and the biopsychosocial model of health [26]. The conceptual model linking PTEs to poor oral health (Fig. 1) illustrates how biological (e.g., chronic stress responses) and psychological (e.g., dental anxiety, maladaptive coping) mechanisms within a broader social context (e.g., healthcare utilisation, socioeconomic factors) contribute to poor SROH. The psychological alterations, dental anxiety and risk behaviours are emphasised (solid line) due to their relevance to this current study. This visual representation of the interactions frames the hypotheses and guides the selection of covariates for the analyses and the interpretation of findings in the current study. Notably, this study focuses on associations and does not conduct mediation or moderation analyses exploring causal pathways and mechanisms.

### Objectives

The study aimed to explore the associations between cumulative exposure to PTEs, adverse dental experiences, and SROH, and to examine how the type of PTE (interpersonal vs. impersonal) and the timing of exposure (before or after age 18) influence these associations.

It was assumed that a higher number of PTEs would be associated with poorer SROH, independent of adverse dental experiences, emotional distress, dental anxiety, oral health behaviours, and sociodemographic factors.

## Methods

### Study design and setting

This observational study used cross-sectional questionnaire data from the Tromsø Study, a population health survey in Tromsø, Norway [27]. Data from the seventh survey (Tromsø 7), conducted from 2015 to 2016, were used [28]. The full questionnaires can be found on the Tromsø Study’s homepage [29].

### Participants

All residents above the age of 40 in Tromsø municipality were invited to participate. There was a 65% response rate, which resulted in 21,069 participants. Of these, 47.5% were men and 52.5% were women. The highest attendance rate was in the age groups 65–69. The socioeconomic profile of participants versus non-participants showed that inhabitants with lower education, lower incomes or living in low-socioeconomic areas were less likely to attend [30]. The most frequently reported PTEs among participants were a life-threatening illness or serious accident of a loved one (36.8%) or of the respondent (24.0%) and bullying (21.5%) [31].

Participants in Tromsø 7 provided written consent for data use, with ethical approval from REK Nord (2018/503282). Consent forms and study details are available on the Tromsø Study website [32].

### Measures

#### Self-reported oral health (SROH)

Global SROH measures predict clinical outcomes like tooth loss and well-being, making them valuable indicators of oral health outcomes [33, 34, 35]. This study employed the Norwegian translation of Locker’s [36] validated one-item measure, “How would you rate your own dental health?” with responses on a five-point Likert scale from “Very poor” (1) to “Excellent” (5). “Dental health” was translated to “oral health” to align with international terminology, a common practice as “dental health” typically reflects overall oral health in self-assessments, especially when translating across languages [36].

#### PTEs

1. Participants were asked to indicate whether they had experienced any of the 11 PTEs listed below, with response alternatives indicating the event’s timing. Three alternatives were available: before age 18, after age 18, or in the last year. Neglect: Neglect during childhood, such as insufficient food, clothes, protection, or love from parents or caregivers.
2. Violence: Been subjected to violence (e.g., beaten, kicked, robbed, or threatened with a firearm).
3. Sexual abuse: Been subjected to sexual abuse, that is, subjected to sexual acts without your consent.
4. Bullying: Been called something negative, excluded, threatened, or harassed by classmates, fellow students, or colleagues over an extended period.
5. Witnessing someone close to you being subjected to violence or sexual assault (e.g., beaten, kicked, beaten up, robbed, threatened with a firearm, or killed).
6. Loss: The death of someone close to you and having difficulty accepting the loss, longing for the deceased, and experiencing intense emotional pain related to the loss.
7. Illness or accident: A life-threatening disease or a serious accident (e.g. fire, work-related or car accident).
8. Adverse hospital event: Received painful or frightening medical treatment while in the hospital because you were sick or seriously injured.
9. Someone close to you has been critically ill or involved in a life-threatening accident (e.g., fire, work, or car accident).
10. Frightening: Experienced something else that was frightening, dangerous, or violent (e.g., natural disaster, war, acts of terrorism, being held captive).
11. Adverse dental event: Received painful or frightening treatment at the dentist.

#### Dental anxiety

The Modified Dental Anxiety Scale (MDAS) was used to measure dental anxiety. This validated self-report questionnaire consists of five items, each rated on a five-point Likert scale (1 = not anxious, 2 = slightly anxious, 3 = Fairly anxious, 4 = Very anxious, and 5 = extremely anxious). Total scores range from 5 to 25, with higher scores indicating greater anxiety. The MDAS had a Cronbach’s alpha of 0.93 (*N* = 20,197).

#### Emotional distress

Emotional distress was assessed using the Hopkins Symptom Checklist (HSCL-10), a validated scale for measuring symptoms of depression and anxiety [37]. Participants rated their experiences over the past week using the response options Not at all, A little, Quite a bit, and Extremely, which yielded scores from 1 to 4, where higher mean scores indicate greater emotional distress. The Cronbach’s alpha of the HSCL-10 was 0.88 (*N* = 19,824).

#### Sociodemographic characteristics

Age was initially recorded as the year of birth. However, for the purposes of this study, we were only granted access to age group data in 10-year intervals. Thus, age was analysed as a categorical variable with 10-year age bands (e.g., 40–49, 50–59, etc.).

Educational level measures the respondent’s highest level of completed education. The response options are elementary school up to 10 years, vocational education, secondary school or upper secondary education (minimum 3 years), college/university education (less than 4 years), and college/university education (more than 4 years).

Financial situation: The respondent’s self-assessed financial situation was measured using a Likert scale in response to the question: ‘How would you evaluate your finances?’ The response options were: very good, good, average, poor, and very poor.

#### Oral health-related behaviour

Teeth brushing frequency was assessed by asking respondents how often they usually brush their teeth, with response options of once a week or less, a few times a week, once daily, and twice a day. Cigarette use was assessed by indicating one out of three categories: current daily smoker (2), previous daily smoker (1) or never daily smoker (0).

The dental visiting pattern was assessed by asking, “Do you regularly visit the dentist or dental hygienist?” The following response options were available: 1: “Yes, more than once a year”, 2: “Yes, annually”, 3: “Yes, every other year”, 4: “Yes, but with longer intervals than two years”, 5: “No, only for acute problems” and 6: “No, I never go”.

### Statistical analysis

Analyses were conducted using IBM SPSS Statistics version 29.0.2.0 [37].

In the bivariate analyses, the responses for SROH were categorised into ‘Poor’ (1 + 2), ‘Average’ (3), and ‘Good’ (4 + 5). As the outcome variable in the regression analyses (described below), the SROH variable was dichotomised into ‘Poor’ (1 and 2) and ‘Good’ (4 and 5), while ‘Average’ (3) was excluded due to inconsistent associations with some covariates. Sensitivity analyses were conducted, where logistic regression models comparing ‘Average’ vs. ‘Good’ oral health and ‘Poor’ vs. ‘Average’ oral health produced similar results, confirming the validity of the findings.

In the first regression analysis, exposure to PTEs was calculated by summing all affirmative responses before age 18, after age 18, and in the last year, resulting in a score ranging from 0 to 24. To distinguish the effects of PTEs outside the dental setting, adverse dental events were analysed separately from other PTEs in this analysis. Quadratic and cubic terms of the PTEs sum score were tested in the regression but were insignificant (*p* = 0.10 and *p* = 0.50). Thus, the PTEs sum score was used as a continuous variable.

The PTEs in the present dataset were closely associated. The two clusters of PTEs identified by Thimm et al. [38], using data from the same sample (Tromsø7), were used as a guide for the four clusters used in the hierarchical regression analyses in this study: Two clusters containing interpersonal events (e.g. violence and sexual abuse) and two clusters containing impersonal events (e.g. life-threatening illness or serious accidents) experienced during childhood and adulthood, respectively [38]. Cluster 1 consists of the following events of interpersonal character that occurred before age 18: bullying, childhood neglect, sexual abuse, violence and witnessed. Cluster 2 consists of the following interpersonal events that occurred after age 18: bullying, frightening, sexual abuse, violence and witnessed. Cluster 3 consists of more impersonal events before age 18: adverse dental events, frightening, adverse hospital events, illness or accident, accident or illness of a loved one and loss. Cluster 4 consists of the following impersonal events occurring after age 18: Adverse dental events, adverse hospital events, accident or illness, accident or illness of a loved one and loss. Thus, the PTEs were grouped according to the four clusters and categorised as 0 = not having experienced any of the PTEs in the cluster and 1 = having experienced at least one of the events in the cluster.

Due to the skewed distribution of the MDAS, with a limited number of individuals reporting high scores, the sum scores were categorised into three levels: ‘low dental anxiety’ (5–10), ‘moderate anxiety’ (11–18), and ‘high anxiety’ (> 19). The high anxiety category corresponds to the established dental phobia cut-off [39]. Introducing a moderate category allowed for a more nuanced understanding of anxiety levels, as the low category permits at most only one ‘extremely anxious’ response, whereas the moderate category permits up to three. This differentiation highlights clinically relevant differences in anxiety severity that are not fully captured by the dental-phobia cut-off.

HSCL-10: In the bivariate analyses, the variable was dichotomised according to the mean sum with a score of 1.85 or higher, indicating emotional distress [40]. Quadratic and cubic terms of the HSCL-10 sum score were tested in the regression but were insignificant (*p* = 0.69 and *p* = 0.53). Thus, the HSCL-10 sum score was used as a continuous variable in the regression analyses.

In the regression analyses, the age groups were divided into ‘middle-aged adults’ (40–59) and ‘seniors’ (60–80). Education levels were categorised as ‘high’: college or university or ‘low’: compulsory and upper secondary. The financial situation was classified as ‘good’: very good and good, ‘average’, and ‘poor’: very poor and poor.

Tooth brushing was dichotomised into ‘twice daily’ and ‘less often than twice daily’. Dental visiting patterns were categorised based on observed associations between visit frequency and oral health parameters in a previous study using the same dataset [41]. The categories were defined as follows: ‘routine visitors’ (those attending annually or every other year), ‘non-routine visitors’ (those attending more than once yearly or less frequent than every other year), and ‘symptomatic/never’ (those who seek care only for acute problems only or never attend), based on associations between visiting patterns and oral health parameters.

Chi-square (χ²) tests were used to examine the associations between SROH, PTEs, and covariates, while effect sizes (Cramér’s V or Phi) were reported to assess the strength of these associations.

Two separate logistic regression analyses were conducted to address the study’s aims. This approach was chosen due to a violation of proportional odds in ordinal regression.

The first logistic regression was used to assess the cumulative effect of PTEs on poor SROH. To isolate the impact of PTEs occurring outside the dental setting, adverse dental events were included as an independent predictor rather than as part of the cumulative PTE score. The estimates were controlled for demographic factors, oral health behaviour, and emotional distress.

The second regression analysis explored the type and timing of PTEs on SROH using a hierarchical logistic regression model. In this analysis, PTEs were grouped into four clusters based on event type (interpersonal vs. impersonal) and timing (before vs. after age 18), as detailed previously. Unlike the first model, adverse dental events were included within the impersonal events category, as described by Thimm et al. [38]. In Model 1, all four PTE clusters were included as independent variables. Model 2 introduced demographic covariates (age, financial situation, and education), Model 3 added oral health-related behaviours (smoking, dental visiting patterns, and toothbrushing frequency), Model 4 incorporated dental anxiety, and Model 5 included emotional distress (measured by HSCL-10).

Linearity of the logit for continuous predictors was tested by including interaction terms between each predictor and its natural log, with non-significant findings confirming the assumption. Multicollinearity was checked using VIF and tolerance values, and no issues were observed. Outliers were not excluded, as they may reflect population variability given the uneven distribution of PTEs. Missing data were handled with pairwise deletion.

## Findings

### Sample demographics characteristics and their associations with SROH

More than half of the participants rated their oral health as good (39.7%) or excellent (15.2%), while 35.2% described it as average. Fewer than 10% considered their oral health poor (7.2%) or very poor (2.7%). Most respondents did not recall any adverse dental experiences. Still, 18.4% reported such an event before age 18, and 5.8% reported one after age 18.

Table 1 presents SROH across categories of PTE exposure, as well as demographic and behavioural characteristics. χ² tests were used to assess whether the associations were statistically significant. Better SROH was associated with fewer PTEs, being female, younger age, higher education, better financial status, non-smoking, frequent toothbrushing, regular dental visits, lower dental anxiety, and lower emotional distress. The strongest associations were found between SROH and dental visits, adverse dental events, dental anxiety, and financial situation.

**Table 1:** Cross-Tabulation of SROH by covariates with Χ² tests

		Self-reported Oral Health (SROH)					
Variables		Good	Average	Poor	Total	χ^2 A^	*Cramer’s V* ^*B*^
∑ PTEs^C^	0	60.5	32.6	6.9	4717		
	1	58.9	33.6	7.5	4727		
	2	55.0	35.7	9.3	3973		
	3	52.8	35.6	11.5	2738		
	≥ 4	47.0	38.4	14.6	4064	285.81*	0.09
Adverse dental events^D^	0	59.1	33.2	7.6	15,519		
	1	42.9	41.2	15.9	4679		
	> 2	32.0	39.3	28.8	219	591.62*	0.17
Sex	Female	58.9	32.6	8.5	10,773		
	Male	50.7	37.9	11.4	9867	146.97*	0.08
Age group	40–49	61.1	30.7	8.1	6410		
	50–59	57.8	33.7	8.5	6002		
	60–69	49.6	39.2	11.2	5072		
	70–79	46.0	40.5	13.5	2502		
	80 +	43.3	40.5	16.1	654	319.13*	0.09
Education	Compulsory	44.3	39.8	15.8	4552		
	Upper Secondary	52.5	37.4	10.1	5695		
	Higher < 4 years	56.8	35.1	8.1	3981		
	Higher > 4 years	64.4	29.6	6.0	6122	565.78*	0.12
Financial situation	Good	61.0	32.0	7.0	14,290		
	Average	43.2	42.6	14.2	5287		
	Poor	28.1	41.1	30.8	725	999.68*	0.16
Daily smoker	Never	63.0	31.2	5.9	8825		
	Previously	52.6	37.2	10.2	8661		
	Current	38.3	41.2	20.5	2742	794.43*	0.14
Toothbrushing	Twice daily	58.1	33.8	8.0	15,971		
	< Twice daily	44.3	40.0	15.8	4230	359.76*	0.13
Dental Visits	> once a year	45.9	41.3	12.7	4319		
	Yearly	64.6	31.1	4.3	10,794		
	Every other year	60.6	33.8	5.6	1899		
	< every other year	44.2	42.9	12.9	1273		
	Only acute	26.5	40.8	32.6	1609		
	Never	23.2	31.1	45.7	315	2420.53*	0.25
MDAS^E^	5–10	58.0	34.4	7.6	16,865		
	11–18	44.0	40.6	15.4	2396		
	≥ 19	25.5	35.5	39.3	575	883.47*	0.15
HSCL-10^F^	< 1,85	56.5	34.8	8.7	17,754		
	≥ 1,85	45.0	37.1	17.8	1694	174.35*	0.10

*p < 0.001

^A^Continuity Correction when df = 1

^B^Phi when df = 1

^C^Potentially Traumatic Events, summarised across lifespan, excluding adverse dental events

^D^ Adverse dental events 1: at least one event reported, 2: adverse events reported both before and after age 18

^E^Modified Dental Anxiety Score

^F^Hopkins Symptom Checklist

### Regression analyses

The regression analyses aimed to investigate the combined, cumulative impact of PTEs and variations in the type (interpersonal or impersonal) and timing of events (before or after 18 years old) of these events on SROH. The baseline log odds in the regression analysis were low, suggesting that the initial probability of reporting poor SROH was low, as most individuals in the population rated their oral health as good.

Table 2 presents the findings of the first logistic regression analysis, with the PTEs sum score and adverse dental events as independent variables. The analysis showed a positive association between adverse dental events and poorer SROH among adults. Additionally, PTEs outside the dental setting were positively associated with poor SROH, independent of adverse dental events. The complete model was statistically significant (χ^2^ (16, *N* = 12124) = 2754.63, *p* < 0.001), indicating that the model could distinguish between respondents who reported poor and good oral health. The model explained 37% (Nagelkerke R squared) of the variance in SROH and correctly classified 88.6% of cases. The goodness of fit was satisfactory according to a Hosmer and Lemeshow test (*P* = 0.41). The result from the regression indicates a small but significant association between the number of PTEs and higher odds of reporting poor SROH. The confidence interval is consistent and falls within a narrow range, indicating the precision of the estimate. Adverse dental events were also significantly associated with a higher likelihood of reporting poor SROH. This effect appeared to accumulate over time, as individuals who reported adverse dental events both before and after the age of 18 had nearly three times higher odds (Odds Ratio (OR) = 2.86, 95% CI:1.75–4.67) of having poor SROH than those who did not report such events. All the covariates included were independently and significantly associated with SROH. The strongest predictors were reporting a symptomatic visiting pattern or never going to the dentist and high dental anxiety, with odds ratios of 9.97 (95% CI: 8.39, 11.86) and 4.59 (95% CI: 3.44, 6.14), respectively. Reporting a poor economic situation, independent of dental anxiety levels and visiting patterns, was associated with more than three times the odds of poor SROH, OR = 3.47 (95% CI: 2.62, 4.61) than those reporting a good economic situation.

**Table 2:** Logistic regression with poor SROH as the dependent variable

Variables^a^	B	SE	OR	CI	
				Lower	Upper
Sum PTEs^b^	0.04^*^	0.02	1.04	1.01	1.08
Adverse Dental Events (df 2)^c^					
At least 1 event (1)	0.59^**^	0.07	1.80	1.56	2.07
Both < 18 & ≥ 18(2)	1.05^**^	0.25	2.86	1.75	4.67
Age: Seniors	0.77^**^	0.07	2.16	1.90	2.47
Sex: Men	0.54^**^	0.07	1.72	1.51	1.96
Financial Situation (df 2)					
Average	0.69^**^	0.07	1.98	1.73	2.27
Poor	1.25^**^	0.14	3.47	2.62	4.61
Education: Lower	0.31^**^	0.07	1.37	1.20	1.56
Smoking Status (df 2)					
Current Smoker	1.09^**^	0.09	2.96	2.48	3.53
Former Smoker	0.40^**^	0.07	1.50	1.30	1.72
Dental Visits (df 2)					
Non-routine visitors	1.11^**^	0.07	3.03	2.65	3.47
Symptomatic/Never	2.30^**^	0.09	9.97	8.39	11.86
Toothbrushing < Twice Daily	0.28^**^	0.07	1.32	1.15	1.53
Dental anxiety^d^ (df 2)					
Moderate	0.71^**^	0.09	2.04	1.71	2.43
High	1.53^**^	0.15	4.59	3.44	6.14
Emotional distress^e^	0.45^**^	0.08	1.57	1.34	1.83
Constant	-4.99^**^	0.14	0.00		

^*^p < 0.05

^**^p < 0.01

^a^Ref. group for categorical variables: Adverse dental events: No painful or frightening dental experiences reported, Sex: female, Age: middle-aged, Economy: Good, Daily smoking: Never, Dental Visits: once every or every other year, Tooth brushing: twice a day, Education: higher (university or college), Dental anxiety: Low (MDAS 5–10)

^b^PTE: Potentially Traumatic Events, not counting adverse dental events

^c^Adverse dental events: 1: at least one event reported, 2: adverse events reported both before and after age 18

^d^MDAS: Moderate Dental anxiety Scale, moderate: MDAS 11–18, high: MDAS ≥ 19

^e^Emotional distress (hscl-10 sum score)

**Fig. 2:**
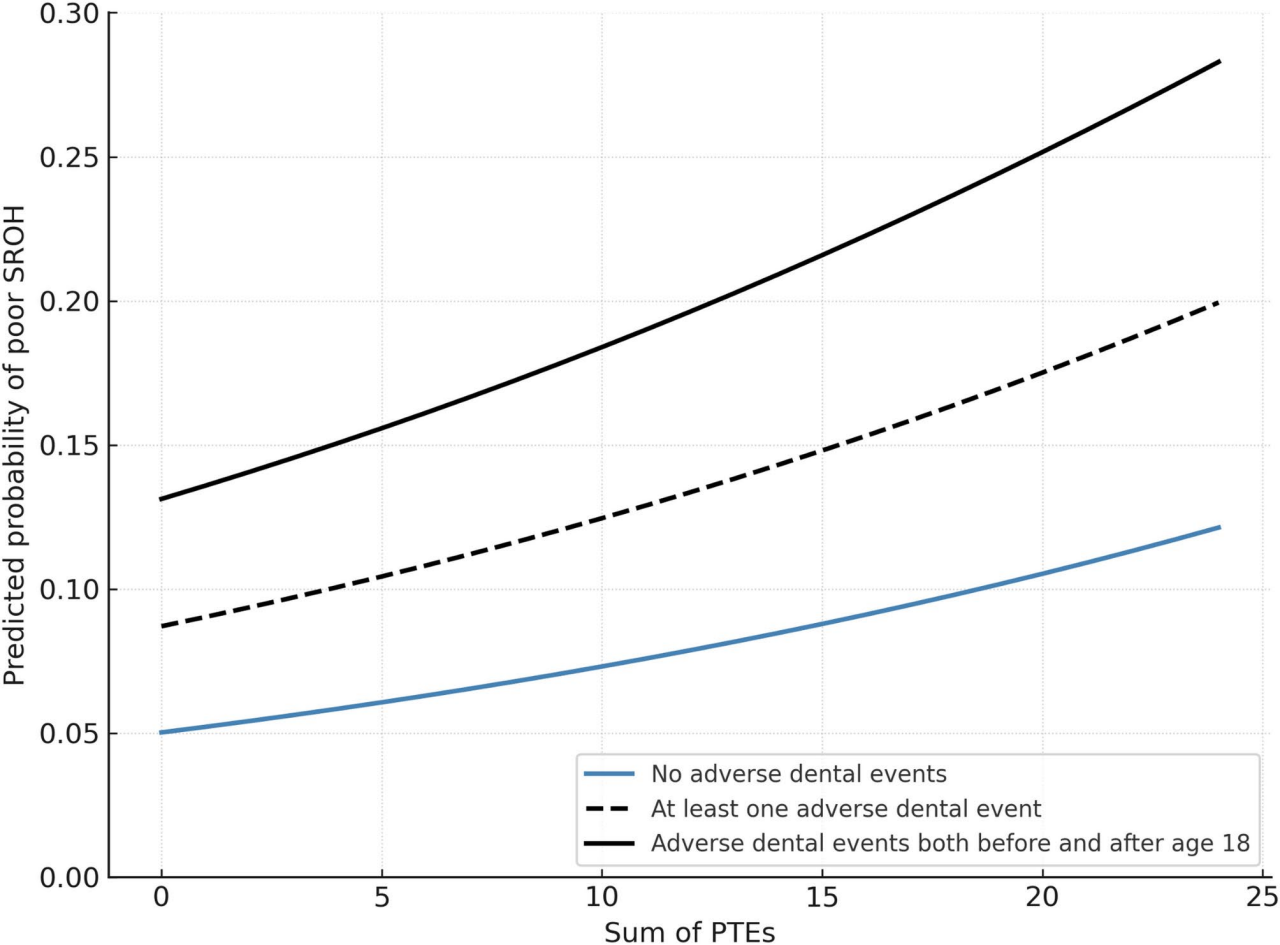
Predicted probability of poor self-rated oral health by cumulative exposure to potentially traumatic events and adverse dental events. The figure illustrates the predicted probability of poor self-rated oral health as a function of cumulative exposure to potentially traumatic events (PTEs), stratified by type of adverse dental events. The figure displays three prediction curves: one for individuals reporting no adverse dental events, one for those reporting at least one event, and one for those reporting events both before and after age 18. Predictions are based on coefficients from the logistic regression model in Table 2. All other variables were held constant at typical or reference values: female sex (reference category), age 40–59 (modal category), higher education, never smoked, dental visits once a year or every other year, good financial situation, low dental anxiety (MDAS score 5–10), and emotional distress (HSCL-10) held at 1.2 (sample median). The model intercept was adjusted upward (from − 4.99 to − 2.94) to improve readability. This affects the overall level of predicted probabilities, but not the relative differences between the adverse dental event categories. The figure shows that greater cumulative exposure to PTEs is associated with a higher probability of poor SROH, especially among individuals with more extensive histories of adverse dental experiences

These relationships are further illustrated in Fig. 2, which presents the predicted probability of poor SROH across levels of PTE exposure stratified by adverse dental events. The figure is based on the logistic regression model (Table 2), with all other covariates held constant at reference or typical values.

The hierarchical regression analysis, detailed in Tables 3a and 3b, revealed that each successive model incrementally improved in explanatory power, with the most substantial increase occurring between Models 1 and 3. Model 4 demonstrated the highest overall explanatory power. For Cluster 1 (interpersonal events before age 18), both the coefficient (B) and OR were slightly but significantly larger with the addition of demographic covariates in Model 2. In contrast, coefficients and odds ratios for the other clusters generally decreased as more covariates were added, though the B coefficient and OR for Cluster 4 remained relatively stable across models.

**Table 3a:** Hierarchical logistic regression of poor SROH: impact of PTEs by timing and type

		Crude effects	Model 1^A^		Model 2^B^		Model 3^C^	Model 4^D^		Model 5^E^	
Cluster 1^I^											
Coefficient (B)		033^**^	0.13^*^		0.17^*^		0.14^*^	0.09		0.02	
Standard Error (S.E.)		0.06	0.06		0.06		0.07	0.07		0.08	
Odds Ratio (OR) with Confidence Intervals (CI)		1.38 (1.24 − 1.54)	1.14 (1.02–1.28		1.18 (1.05–1.34)		1.15 (1.01–1.32)	1.09 (0.95–1.25)		1.02 (0.89–1.18)	
Cluster 2^II^											
B		0.45^**^	0.28^**^		0.28^**^		0.17^*^	0.16^*^		0.10	
S.E.		0.06	0.06		0.07		0.07	0.08		0.08	
OR (CI)		1.56 (1.39–1.75)	1.33 (1.18–1.50)		1.32 (1.16–1.50)		1.18 (1.03–1.37)	1.18 (1.02–1.36)		1.11 (0.95–1.28)	
Cluster 3^III^											
B		0.75^**^	0.61^**^		0.52^**^		0.44^**^	0.31^**^		0.30^**^	
S.E.		0.05	0.06		0.06		0.07	0.07		0.07	
OR (CI)		2.11 (1.90–2.34)	1.85 (1.65–2.07)		1.68 (1.49–1.89)		1.55 (1.36–1.76)	1.36 (1.19–1.55)		1.35 (1.18–1.54)	
Cluster 4^IV^											
B		0.49^**^	0.21^**^		0.21^**^		0.20^**^	0.20^*^		0.18^*^	
S.E.		0.05	0.06		0.06		0.07	0.07		0.07	
OR (CI)		1.63 (1.47–1.81)	1.24 (1.10–1.39)		1.24 (1.01–1.39)		1.22 (1.07–1.39)	1.22 (1.07–1.40)		1.20 (1.05–1.37)	

^*^p < 0.05

^**^p < 0.001

^I^ Interpersonal events experienced in childhood or adolescence (before age 18)

^II^ Interpersonal events experienced in adulthood (after age 18)

^III^ Impersonal events experienced in childhood or adolescence (before age 18)

^IV^ Impersonal events experienced in adulthood (after age 18)

^A^ PTEs in four clusters (df = 4)

^B^ Demographic covariables added in this model: age, financial situation, education (df = 9)

^C^ Oral health-related variables added in this model: smoking, dental visiting patterns, toothbrushing frequency (df = 14)

^D^ Dental anxiety as measured by Modified Dental Anxiety Scale added in this model (df = 16)

^E^Emotional Distress measured by Hopkins Symptom Checklist-10 added in the final model (df = 17)

The addition of covariates had less effect on the association between impersonal events and SROH than on the association between interpersonal events and SROH (Clusters 1 and 2), as indicated by changes in B coefficients and ORs in Tables 3a and 3b. Among all clusters, impersonal childhood events (Cluster 3) consistently showed the highest odds ratios and remained significant across all models, suggesting a robust association with SROH.

Taken together, the findings indicate a positive association between cumulative PTE exposure and poor SROH, independent of adverse dental events, emotional distress, dental anxiety, oral health behaviours, and sociodemographic factors. However, the associations varied when considering different types of PTEs and their timing. Impersonal childhood events consistently demonstrated the strongest and most stable association with poor SROH, even after adjusting for all covariates.

**Table 3b:** Hierarchical Logistic Regression Model Comparison: ∆Χ² and Nagelkerke R-Square Values

	- 2 Log likelihood	∆Χ²	P	Nagelkerke R Square
Model 1	9637.49	249.5	< 0.001	0.04
Model 2	8756.65	880.84	< 0.001	0.16
Model 3	7444.35	1312.31	< 0.001	0.33
Model 4	7191.38	252.97	< 0.001	0.36
Model 5	7156.44	34.45	< 0.001	0.36

## Discussion

This study investigated associations between cumulative exposure to PTEs and SROH. It also examined how different types of PTEs, and the timing of exposure influenced these associations. The findings aligned with the initial expectations, indicating that a higher number of PTEs was associated with poorer SROH when controlling for emotional distress, dental anxiety, oral health-related behaviours and socioeconomic factors. Among the PTEs, adverse dental experiences emerged as the strongest predictor of poor SROH.

The associations between combined PTEs and poor SROH are noteworthy, considering that fewer than ten per cent of participants rated their oral health as poor, while more than half rated it as good. Furthermore, most of the population under study reported socioeconomic and oral health-related behaviours that are beneficial to oral health outcomes. Had the participants included more of the underserved population [30], the impact of PTEs might have been more pronounced. While the effect sizes are small, their cumulative impact in a population context could be considerable, suggesting that minor individual effects can translate into meaningful outcomes on public health.

The findings indicate that timing and type of PTEs are relevant in their impact on poor SROH. In contrast to interpersonal events in childhood and adulthood, impersonal events in childhood or adolescence had a higher impact on poor SROH than those reported in adulthood, suggesting that non-relational stressors during formative years can have long-lasting effects independent of events later in life.

The strong association between poor SROH and impersonal events in childhood is most likely largely attributable to adverse dental events. It seems that these experiences not only influence the development of dental anxiety but might also have an independent direct impact on SROH in adulthood. This association can be interpreted in several ways. First, negative dental experiences in childhood may reflect underlying oral health issues and a greater need for invasive treatments, supporting the view that oral health disparities often originate early in life, particularly given that dental caries is a chronic, cumulative condition. However, reporting adverse dental experiences at the dentist may extend beyond immediate dental treatment needs, pointing to broader vulnerabilities such as social context, lower pain tolerance, temperamental traits, patterns of emotional and behavioural regulation, or other prior adversities not captured by the PTEs in this study, including emotional abuse or household dysfunction.

Adverse childhood experiences are associated with less preventive care and a higher risk of tooth decay [42]. Longitudinal studies show that children with high caries rates, lack of access to community water fluoridation, low socioeconomic status, lower IQ scores, and parents who rate their child’s oral health as poor often follow an unfavourable trajectory, with dental disease accumulating from childhood into adulthood [43]. These patterns underscore the importance of examining risk factors beyond individual oral health behaviours and previous caries experiences. Thus, reports of adverse dental experiences can signal underlying challenges, as children with additional vulnerabilities may perceive and respond to treatment situations differently than those without such challenges. Recognising these complexities is essential for understanding the broader impact of childhood experiences on oral health and for developing preventive strategies that mitigate negative clinical experiences. This indicates that a solid foundation for good oral health in adulthood is established through positive childhood dental experiences. In essence, paying attention to children’s subjective experiences in the dentist’s chair and aiming to provide good experiences can have a lasting positive impact on their oral health.

The relationships involving interpersonal events were more complex. When factors such as dental anxiety and emotional distress were accounted for, the significant influence of interpersonal events on poor SROH diminished. Previous findings have found that interpersonal events, such as sexual abuse and childhood neglect, are partially or completely linked to dental anxiety through their association with emotional distress [16]. Furthermore, among individuals with high dental anxiety, those with a history of abuse tend to experience more psychological symptoms of anxiety and poorer oral health-related quality of life than those without such a history [44]. Recognising the difficulties that abuse survivors encounter in accessing dental care, alongside the influence of adverse childhood experiences on pain perception, indicates that children with trauma histories may be particularly vulnerable to negative dental encounters. Consequently, childhood interpersonal trauma could heighten susceptibility to pain and feelings of helplessness during dental treatment. Integrating assessments of both PTEs and previous dental experiences in patient evaluations may be essential for delivering supportive and trauma-informed care in clinical settings.

This study confirms socioeconomic disparities in oral health [45]. In Norway, limited access to dental care is often due to personal financial constraints, as most adults must cover these expenses out of pocket, with public subsidies primarily reserved for specific groups, including children, adolescents and young adults. Even after adjusting for dental visiting patterns and educational level, the study found that a poor financial situation is strongly linked to poor SROH. To effectively address these disparities, political initiatives are needed to make economic status and education irrelevant to health outcomes. This includes making healthy behaviour choices more accessible.

For interpersonal events in childhood, the findings in this study indicate that the relationship with poor SROH appears to be masked by factors like current financial status, education, and age. When these factors are added to the models, the independent association of childhood interpersonal events on poor SROH strengthens, and the models significantly improve. In other words, poor socio-economic status and interpersonal PTEs in childhood seem to have compounding effects in this study, with both contributing to poor SROH. These factors are also interrelated; socioeconomic status and adverse childhood experiences often interact in complex ways. For example, children from lower socioeconomic backgrounds are more likely to experience PTEs [46]. Research has shown that these experiences can disrupt learning and school attendance [47], leading to lower academic achievement and reduced educational attainment. Lower educational attainment often results in limited job opportunities and lower income levels in adulthood. Furthermore, the link between economic disadvantage and educational achievement can persist across generations [48]. In addition to the effects of PTEs, economic constraints may limit access to regular oral care and preventive services, contributing to poorer oral health outcomes across the lifespan [49]. Addressing the root causes of PTEs and supporting affected families can help break the cycle of disadvantage and improve oral health outcomes.

Health behaviours are one of several important pathways linking PTEs to poor health outcomes [50]. Individuals who experience PTEs in childhood may adopt unhealthy coping mechanisms, such as smoking [51], which can negatively impact oral health. Adversities in childhood and lower educational attainment can reduce health literacy, making it harder for individuals to understand and adopt healthy behaviours [52, 53]. The analyses underline the adverse effects of smoking, infrequent toothbrushing and sporadic or avoidance of dental health services on SROH. The fact that the strength of the associations across all groups decreases when behavioural factors are included (Table 3a) suggests that these behaviours may partly explain the link between PTEs and poor SROH.

The current study supports an association between emotional distress and an increased risk of poor SROH, a finding supported by other population studies [54]. With each one-point rise in emotional distress, the likelihood of reporting poor SROH was 57% higher. This emphasises the strong connection between mental health symptoms and oral health.

Although the associations identified in this study do not imply causality, they highlight the need for trauma-sensitive approaches in dental care. Recognising that adverse dental experiences in childhood are strongly associated with poor SROH in adulthood, clinicians should prioritise creating positive treatment experiences early in life.

Integrating trauma-informed care into dental practice, particularly in pediatric settings, may help prevent long-term consequences of dental anxiety and care avoidance. Screening for previous negative dental experiences and tailoring patient interactions accordingly could improve treatment adherence and promote more equitable oral health outcomes. Public oral health policies should also align with broader health strategies addressing the psychological burden of early-life adversities. Expanding access to mental health support within dental services and improving awareness of dental anxiety in professional training could contribute to reducing disparities in oral health.

### Limitations

When interpreting the findings of this study, several limitations must be considered.

This study’s cross-sectional design limits the ability to draw causal inferences about the relationships between PTEs and SROH. Longitudinal studies are needed to elucidate the pathways through which PTEs affect oral health outcomes and to identify protective factors that may mitigate these effects over time. Retrospective reports of PTEs are also prone to recall bias [55].

Although the sample size is large, the population is not sociodemographically representative, and response rate bias might have influenced the findings [30].

Another limitation of this study is the translation of self-reported “dental health” from Norwegian to self-reported “oral health” in English. While the term “dental health” is often used more broadly in everyday language, aligning more closely with the concept of “oral health” as it is applied in professional contexts, individual interpretations of these terms may vary. These linguistic nuances should be considered when interpreting the findings, especially when comparing with studies that use the term “oral health”. Moreover, interpreting self-reported health measures is inherently complex, as they reflect more than just clinical diagnoses. Subjective characteristics like age, dental anxiety and aesthetics can influence how individuals perceive their oral health.

While this study did not include clinical oral health data, self-reported measures are well established as meaningful indicators of perceived oral health and treatment needs. Previous research has shown that such measures remain stable over time and capture relevant dimensions for service planning [56]. Combining subjective and clinical indicators would likely offer a more comprehensive understanding of oral health outcomes.

This study’s findings are based on the adult population in Tromsø, Norway. Therefore, caution should be exercised when transferring these findings to other populations with different sociodemographic characteristics, cultural backgrounds, and healthcare systems. To enhance their generalisability, further studies are needed to validate these findings in diverse populations.

## Conclusion

This study provides evidence of an association between PTEs across different life stages and SROH in adulthood. Among all potentially traumatic experiences assessed in this study, adverse dental experiences during childhood showed the strongest association with poor self-reported oral health.

Addressing dental treatment’s emotional and psychological aspects, particularly in paediatric care, seems crucial for improving patients’ long-term oral health outcomes. Public oral health policies should incorporate trauma-informed approaches in dentistry and oral health strategies should be aligned with broader mental health initiatives. Integrating psychological support into dental care and improving awareness of dental anxiety in healthcare settings may help reduce oral health disparities.

## Data Availability

Researchers affiliated with approved research institutions can apply for access to research of the Tromsø Study’s material, available from The Tromsø Study [https://uit.no/research/tromsostudy]. All projects applying for data in the Tromsø study must have their own approval from the Regional Ethics Committee (REK).

## Abbreviations

HSCL-10: Hopkins symptom checklist
MDAS: Modified dental anxiety scale
PTE: Potentially traumatic event
SPSS: IBM statistical package for the social sciences
SROH: Self-reported oral health
χ²: Chi-squared
OR: Odds ratio
